# BLADDER TRANSPLANTATION: THE NEW FRONTIER IN ABDOMINAL ORGAN TRANSPLANTATION

**DOI:** 10.1590/0102-6720202400015e1808

**Published:** 2024-06-17

**Authors:** Affonso Celso PIOVESAN, Wellington ANDRAUS, Anderson Bruno PELLANDA, Elias DAVID, Luiz Carneiro D´ALBUQUERQUE, William Carlos NAHAS

**Affiliations:** 1 Universidade de São Paulo, Hospital das Clínicas, Renal Transplant Unit – São Paulo (SP), Brazil; ^2^Universidade de São Paulo, Hospital das Clínicas, Digestive Transplant Unit – São Paulo (SP), Brazil.; 2 Universidade de São Paulo, Hospital das Clínicas, Digestive Transplant Unit – São Paulo (SP), Brazil

**Keywords:** Transplantation, Urinary Bladder, Kidney, Cystectomy, Transplante, Bexiga Urinária, Rim, Cistectomia.

## Abstract

Lower urinary tract abnormalities are directly implicated in the etiology of renal dysfunction in 6 to 24% of dialytic patients. These patients require bladder capacity and compliance readjustment before being considered viable candidates for renal transplantation. Vesical augmentation surgeries often involve the use of intestinal segments. Although these procedures can effectively restore bladder capacity and compliance, they present various issues related to maintaining mucous absorption and secretion capacity. Acidosis, recurrent urinary tract infections, and stone formation are extremely common, leading to frequent hospitalizations and graft function loss. Urinary tissue is certainly ideal for these reconstructions; however, bladder augmentation using ureter and renal pelvis are feasible only in a minority of cases. Experimental studies have been conducted to establish the groundwork for vascularized bladder transplantation. Last year, for the first time, this procedure was performed on a brain-dead patient. During this intervention, cystectomy was performed with preservation the vascular pedicle, followed by organ reimplantation. The graft remained viable for a period of 12 hours post-transplant. However, this intervention utilized a robotic platform, making it less reproducible in a multi-organ procurement setting as well as for most transplant centers. Moreover, it is debatable whether the benefits of exclusive bladder transplantation outweigh the risks associated with immunosuppression. For patients needing renal transplantation and requiring lower urinary tract reconstruction, however, utilizing the donor’s bladder may offer an attractive alternative, avoiding the inherent complications of enterocystoplasty without increasing immunological risk. Combined kidney and bladder transplantation has the potential to emerge as the next frontier in abdominal organ transplants.

## RESUMO

As alterações do trato urinário inferior estão diretamente implicadas na etiologia da disfunção renal em 6 a 24% dos pacientes em diálise. Esses pacientes necessitam readequação da capacidade e complacência vesical antes de serem considerados candidatos viáveis para o transplante renal. As cirurgias de ampliação vesical frequentemente envolvem a utilização de segmentos intestinais. Embora estes procedimentos possam reestabelecer de forma eficaz a capacidade e complacência vesical, apresentam diversos problemas relacionados à manutenção da capacidade de absorção e secreção de muco. Acidose, infecções urinárias de repetição e formação de cálculos são extremamente comuns levando a internações frequentes e perda de função do enxerto. O tecido urinário é certamente ideal para estas reconstruções, contudo, ampliações vesicais utilizando ureter e pelve renal são viáveis somente em uma minoria dos casos. Estudos experimentais têm sido conduzidos na busca de se estabelecer os fundamentos para um transplante vascularizado de bexiga. No ano passado, pela primeira vez, este procedimento foi realizado em um paciente em morte encefálica. Nessa intervenção, foi realizada a cistectomia, preservando-se o pedículo vascular, seguida pelo reimplante do órgão. Esse enxerto mostrou-se viável pelo período de 12 horas após o transplante. Entretanto, nesta intervenção, foi utilizada plataforma robótica tornando-o pouco reprodutível em um contexto de captação de múltiplos órgãos bem como para a maioria dos centros transplantadores. Além disso, é discutível se os benefícios do transplante vesical exclusivo compensam os riscos associados à imunossupressão. Para pacientes que precisam ser submetidos a transplante renal e requerem reconstrução do trato urinário inferior, entretanto, a utilização da bexiga do mesmo doador pode representar uma alternativa atraente, evitando as complicações inerentes às enterocistoplastias sem aumento do risco imunológico. O transplante combinado de rim e bexiga tem o potencial de se destacar como a próxima fronteira nos transplantes de órgãos abdominais.

## INTRODUCTION

A multitude of conditions can compromise bladder integrity, leading to a loss of the organ’s capability to store urine at low pressures. These conditions frequently contribute to renal function deterioration and the subsequent need for renal replacement therapy. In the United States, urinary tract anomalies are implicated as a causative factor in renal function loss in approximately 6% of kidney transplant recipients^9^. This incidence escalates to 24.1% in pediatric patients due to congenital anomalies affecting the lower urinary tract, such as myelomeningocele, posterior urethral valves, and spina bifida^24^. Similarly, in Brazil, 35% of transplanted children have urological anomalies as the primary etiology of renal function loss^17^. While kidney transplantation is acknowledged for its cost-effectiveness and enhancement of patient survival and quality of life compared to dialysis, a prerequisite for successful renal transplantation in these patients is the optimization of the lower urinary tract to prevent compromise to the graft, as observed in native kidneys^11,21^.

The inaugural urinary diversion utilizing an intestinal segment was executed by Von Mikulicz in 1889^25^, with routine application following Couvelaire’s publication in 1950^7^. It was not until two decades later that the first kidney transplants in patients with urinary reconstructions involving intestinal segments were reported^16,22^. Presently, bladder capacity augmentation through an ileal segment is the most commonly employed method for such reconstructions. Despite its effectiveness in increasing capacity and compliance, the intestinal segment continues to absorb urinary toxins, potentially leading to acidosis and premature dialysis requirement due to its absorptive function^5^. Additionally, the maintenance of mucus secretion contributes to the formation of urinary stones, with an incidence ranging from 14 to 52%^27^.

Furthermore, these patients frequently experience bacteriuria and urinary tract infections, with incidences reported between 50 to 70%^9^. Among the 116 patients who underwent kidney transplantation after enterocystoplasty at our institution, 79% experienced recurrent pyelonephritis.

Long-term exposure of urine to intestinal mucosa may induce malignant transformations, culminating in adenocarcinoma development. Enlarged bladders with an ileal segment present a tumor incidence of about 5%, often manifesting years post-procedure^3^. While the urinary tract tissue remains the ideal medium for bladder augmentation, the use of remodeled ureter, pelvis, and calyces as a bladder flap is infrequent, due to typically insufficient capacity^18^. Several alternative methods for bladder expansion have been explored, but none have yet become standard practice due to less than satisfactory results^1,2^.

The concept of using a non-pedicled bladder flap sutured to the recipient’s bladder was first reported by Calzada from the University of Malaga in 1987^14^. Subsequent isolated case reports have utilized non-pedicled bladder grafts to facilitate ureteral reimplantation, rather than to increase storage capacity^8,12,15,20^. More recent experimental studies have investigated pedicled bladder grafts, such as the combined kidney and bladder transplant in swine conducted by Torino from the University of Rome in 2013^23^. However, these have encountered varying success rates, with some animals requiring exploratory laparotomy within days due to graft thrombosis, presumably from rejection^26^.

To lay the groundwork for human bladder transplantation, cadaveric models were also developed to elucidate organ perfusion and possible anatomical variations, with a focus on arterial irrigation^13^. In 2023, Nassiri and Gill conducted the first human pedicled bladder transplantation in a non-organ-donation candidate brain-dead patient after extensive pre-clinical studies in pigs and pulsatile cadavers. The procedure included robotic cystectomy, bench graft preparation, and robotic implantation, with successful graft perfusion observed during a 12-hour surgical exploration^19^.

Despite this pioneering human intervention, the robotic harvesting route presents challenges, rendering it impracticable for most transplant services due to extended surgical times and the logistical demand for immediate robotic technology access, compounded by the complexity of multidisciplinary organ retrieval efforts. Furthermore, robotic organ reimplantation is beyond the technical capabilities of many surgeons, constraining the number of teams capable of conducting such transplants.

Considering the relative satisfaction with enterocystoplasty outcomes despite their complications, the net benefit of bladder transplantation may not justify the associated immunosuppression risks. Nevertheless, in cases requiring both procedures, kidney transplantation and bladder enlargement, the utilization of bladder tissue from the digestive system could be circumvented without additional immunosuppression risks. Utilizing kidneys and bladders from the same deceased donor and performing both transplants simultaneously should not alter antigen exposure; thus, no additional immunological impact on renal transplant success is anticipated. Additionally, this approach spares the patient from undergoing two separate surgical procedures and reduces dialysis duration by eliminating the wait for bladder enlargement and recovery before kidney transplant eligibility.

From an immunological perspective, the bladder, predominantly composed of muscle tissue and devoid of lymphoid structures, is hypothesized to exhibit a rejection profile akin to cardiac grafts, which require lower immunosuppression doses compared to kidney grafts. Consequently, in combined kidney and bladder transplants, immunosuppression regimens may not necessitate modification.

## CONCLUSIONS

In summary, dual kidney and bladder transplantation presents a logical intervention for patients on renal replacement therapy with neurogenic bladder who require bladder augmentation prior to kidney transplantation. The successful transplantation of a uterus from a deceased donor, another muscular pelvic organ, corroborates the feasibility of this approach^4,6,10^. Bladder transplantation, in conjunction with kidney transplantation, may represent the next frontier in abdominal organ transplantation.
